# The association between methylation patterns of DNAH17 and clinicopathological factors in hepatocellular carcinoma

**DOI:** 10.1002/cam4.1930

**Published:** 2018-12-21

**Authors:** Xiaoxiao Fan, Hongbin Guo, Binghua Dai, Lifeng He, Daizhan Zhou, Hui Lin

**Affiliations:** ^1^ Department of General Surgery, Sir Run Run Shaw Hospital, School of Medicine Zhejiang University Hangzhou China; ^2^ Biomedical Research Center, Sir Run Run Shaw Hospital, School of Medicine Zhejiang University Hangzhou China; ^3^ Department of Neurosurgery, Xia Sha campus, Sir Run Run Shaw Hospital, School of Medicine Zhejiang University Hangzhou China; ^4^ The Department of liver transplantation and Special Treatment, Eastern Hepatobiliary Surgery Hospital Second Military Medical University Shanghai China

**Keywords:** clinicopathological factors, *DNAH17*, hepatocellular carcinoma, methylation

## Abstract

**Background:**

Hepatocellular carcinoma (HCC) is a malignancy with poor prognosis. Complex genetic and epigenetic alterations are the two primary causes of HCC. The aim of the study was mainly to explore the correlation between the methylation status of *DNAH17* and HCC.

**Methods:**

We evaluated the methylation levels of *DNAH17* in 163 HCC samples and their paired normal tissue using Sequenom EpiTYPER assays and performed the TaqMan copy number assay to assess the copy number status of *DNAH17* in HCC samples.

**Results:**

The mean methylation levels were significantly decreased in the tumor tissues compared to the paired normal tissues in both selected regions of *DNAH17* (amplicon 1:58.7% vs 84.5%, *P* < 0.0001; amplicon 2:69.9% vs 84.5%, *P* = 0.0060). Contrarily，both RNA‐seq and immunohistochemistry indicated the expression of *DNAH17* was increased in tumor tissues (*P* < 0.05). DNMT inhibitor decitabine treatment could increase the expression of *DNAH17* in HCC cell lines. *DNAH17* gene amplification always companied with hypomethylation status. Moreover, hypomethylation status was associated with several clinical characteristics, such as male patients, higher AFP values, higher age of onset, fibrous capsules, tumor necrosis, liver cirrhosis, and tumor thrombus (*P* < 0.05). Receiver operator characteristic (ROC) curve analysis demonstrated the methylation levels of *DNAH17* could efficiently predict the existence of the fibrous capsule (AUC = 0.695) and tumor thrombus (AUC = 0.806).

**Conclusions:**

These findings suggested that aberrant methylation of *DNAH17* was associated with comprehensive HCC clinicopathological factors and could be a promising biomarker for tumor thrombosis in HCC patients.

## 1 INTRODUCTION

Hepatocellular carcinoma (HCC), accounting for 70%‐90% of all primary liver cancers, is one of the leading causes of cancer death, especially in less‐developed countries.^1^ Although many new therapeutic methods have been introduced, and surgical techniques are being developed rapidly, the outcomes of HCC treatment have not improved notably. Few patients have an opportunity to receive curative treatment, either primary resection or liver transplantation, because many patients were initially diagnosed at an advanced stage. Tumor thrombosis is one of common features of the advanced stage HCC and indicates a poor prognosis.^2^, ^3^ Therefore, it is crucial to explore underlying mechanisms of HCC initiation and progression and find more accurate biomarkers, for example, for tumor thrombosis, to help clinicians draw a precise therapeutic strategy.

Currently, aberrant methylation of tumor oncogenes and tumor suppressor genes has been noted in many cancers, leading to expression changes of cancer‐related genes that promote tumorigenesis and progression.^4^, ^5^ Solid evidence revealed the significance of methylation deregulation in HCC.^4^, ^6^, ^7^

**Figure 1:**
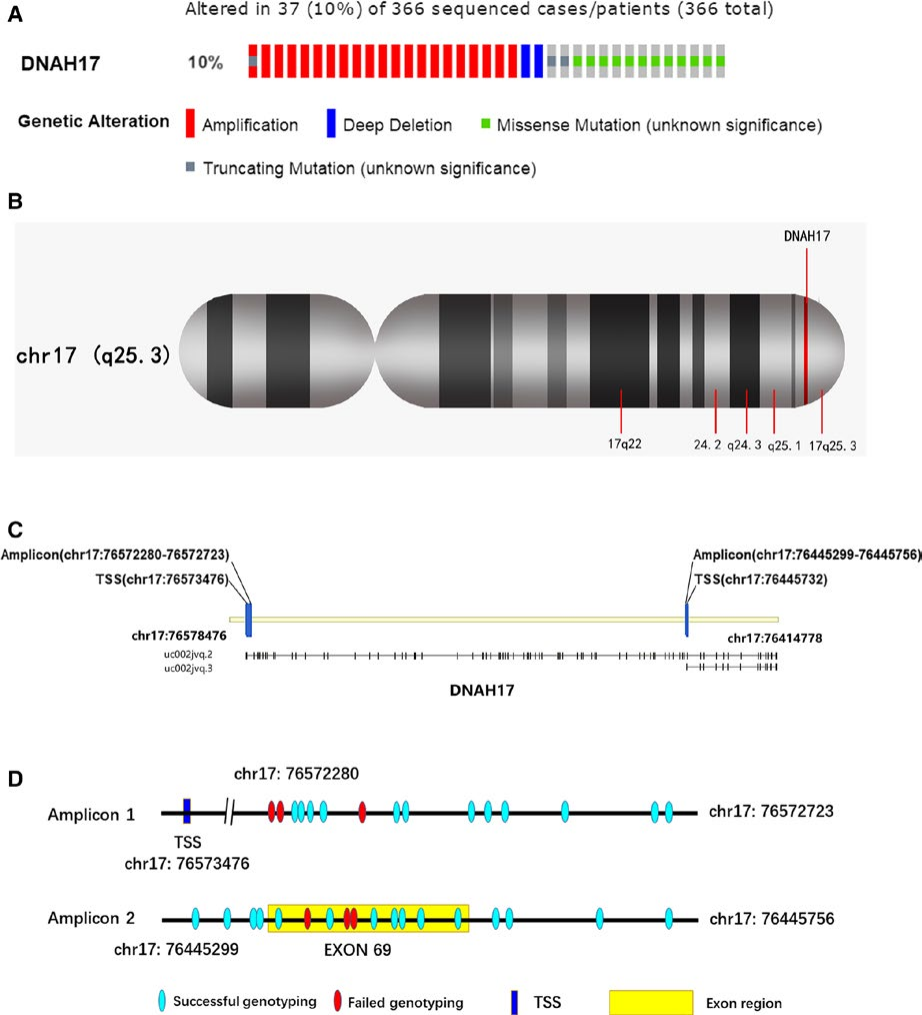
The basic genetic information of* DNAH17*. A, Schematic diagram of the *DNAH17* genetic alterations from cBioPortal website (http://www.cbioportal.org/). B, The gene location in the genome. C, The locations of amplicon 1 and amplicon 2 in *DNAH17*. D, The location of CpG sites in each amplicon

Dynein, which is composed of several light, heavy, and intermediate chains, is an essential protein for the primary cilia. Davey et al^8^ reported that the primary cilia were widely observed in the developing liver. Unlike the motile cilia, the primary cilia are microtubule‐based organelles located at the cytomembrane that act as sensors to receive physical and chemical signals.^9^ In the past ten years, this largely ignored organelle has been brought to the forefront of cancer research.^9^, ^10^, ^11^, ^12^ Loss of cilia was observed in various cancer cells and gradually regarded as a common hallmark of the disease.^9^, ^11^, ^12^, ^13^ Restoring the expression of primary cilia appears to be a promising therapeutic method for cancer treatment.^14^, ^15^ However, the detailed mechanism of primary cilia in oncogenesis and tumor progression remains poorly understood. Dynein axonemal heavy chain 17 (DNAH*17*), which was initially mapped in 1998,^16^ is a gene encoding a heavy chain associated with axonemal dynein. Recently, whole‐exon sequencing of five paired hepatitis B virus‐associated early‐stage HCC samples found that *DNAH17* was frequently mutated in HCC patients.^17^ The data from the Cancer Genome Atlas (TCGA) dataset confirmed the conclusion that genetic alteration of *DNAH17* was common. A total of 186 (51%) of 366 sequenced HCC patients had genetic alteration in dynein axonemal heavy chain genes, and *DNAH17 *was found to be altered in ten percent of all sequenced patients (Figure 1A); it is one of the top two most frequently altered genes among all DNAH genes. This interesting finding encouraged us to explore the potential relationship between *DNAH17* and hepatocellular carcinoma.

In this study, we conducted Sequenom EpiTYPER assays to investigate the potential mechanism and the correlation between methylation of *DNAH17* and clinicopathological features. We found that overexpression of *DNAH17* by down‐regulation of methylation levels might contribute to HCC initiation and progression. In addition, the hypomethylation status of the *DNAH17* gene, both in tumor tissue and adjacent non‐cancerous tissue, could be a promising biomarker for tumor thrombosis in HCC.

## 2 MATERIALS AND METHODS

### 2.1 Patients and samples

Hepatocellular carcinoma tissues and paired adjacent non‐cancerous tissues were collected from 163 patients receiving curative resection at the Shanghai Eastern Hepatobiliary Surgery Hospital from January 2012 to December 2012. All samples were immediately frozen in liquid nitrogen and stored at −80°C until DNA was extracted. According to the TNM staging system of the American Joint Committee on Cancer (AJCC 7th edition), all the patients were diagnosed as HCC, and the pathological features were assessed by two pathologists.

A total of 138 male patients and 25 female patients were included in our study. The average age was 51.6 ± 10.3 years old (Mean ± Standard Deviation). Patients’ personal information, family history, clinical testing results, pathological information, and other data were collected in accordance with hospital privacy rules. This study protocol was approved by the Clinical Research Ethics Boards of the Shanghai Eastern Hepatobiliary Surgery Hospital, and informed consents were obtained from all patients.

### 2.2 Cell culture and decitabine treatment

Two HCC cell lines, Huh7 and PLC/PRF/5, were selected for decitabine treatment. The cell lines were kindly provided by Cang's lab (Zhejiang University, Hangzhou, China). They purchased the cell lines from the Type Culture Collection of the Chinese Academy of Sciences (Shanghai, China) (Huh7 Cat# 12800017, PLC/PRF/5 Cat# 41500034). Cell lines were cultured in DMEM (Gibco, Cat# C11995500BT) with 10% fetal bovine serum (FBS) (Cellmax, Cat# SA102.02) and maintained at 37°C in 5% CO2. Decitabine, a DNMT inhibitor, was purchased from Targetmol (Cat# T1508). For decitabine treatment, cells were pre‐cultured to 10%‐20% confluence and then cultured with medium containing decitabine for 72 hours at different doses. Two concentrations of decitabine, 5 and 10 μmol/L, were used for treatment. The decitabine was diluted in dimethylsulfoxide (DMSO) (Sigma, CAT# D2650). In the control group, we added the same volume of DMSO. The medium was refreshed every day. The decitabine treatment experiments were repeated for three times in both two HCC cell lines.

### 2.3 RNA extraction and real‐time quantification PCR

Total RNA was isolated using Trizol reagent (Invitrogen, Carlsbad, CA, USA) according to the specification, and 1 μg RNA was then reversely transcribed to cDNA with Hifair™ 1st Strand cDNA Synthesis Kit (Yeasen, Shanghai, China, Cat# 11123ES10). Quantitative real‐time PCR was performed with Hifair™ qPCR SYBR Green Master Mix (Yeasen, Cat# 11201ES08). qRT‐PCR was performed by a 7500 RT‐PCR system (Thermo Fisher Scientific, Waltham, MA, USA), and the annealing temperature was 55°C. GAPDH served as a normalizing control. The qPCR primers for *DNAH17*, forward primer, 5′‐TTACACCAACGTCACTGAAGGG‐3′ and reverse primer, 5′‐AGTCGGCTTGTTCCATCTCCT‐3′; for GAPDH, forward primer, 5′‐GTGAAGCAGGCGTCGGA‐3′ and reverse primer, 5′‐AGCCCCAGCGTCAAAGG‐3′. The range of the obtained Ct values was 15‐30. Each sample was tested in triplicate. The 2^−ΔΔCT^ as a calculation method was performed to analyze the expression of *DNAH17* gene in the selected cell lines after decitabine treatment.

### 2.4 Immunohistochemistry (IHC)

The 20 pairs of HCC samples and their paired normal liver tissues were selected for immunohistochemistry.The paraffin‐embedded tissue blocks were obtained from the department of pathology and sectioned into 3‐μm‐thick slides. Then, the slides were deparaffinized and rehydrated using xylene (Yonghua Chemical Technology, Jiangsu, China, Cat# 155502104) and ethanol (Yonghua Chemical Technology, Jiangsu, China, Cat# 117902104). The slides were further immersed in 10 mmol/L citrate buffer (pH 6.0, Meilunbio Cat# MA0180) and boiled for 10 minutes in an autoclave. The endogenous peroxidase activity was blocked with 3% hydrogen peroxide (Yonghua Chemical Technology, Cat# 210402104) for 5 minutes. 5% normal goat serum (Meilunbio, Cat# MB4508) was used to block the non‐specific binding for 30 minutes. For *DNAH17* expression analysis, the sample slides were immunostained with the anti‐*DNAH17* antibody (R&D System, Cat# MAB9657‐SP) overnight at 4°C in 1:200 dilution and then stained with hematoxylin (Meilunbio, MB9897) for 2 minutes. The GTVision III detection system (Gene Tech, Shanghai, China, Cat# GK500710) was used for detecting the expression of *DNAH17*, and we assessed the immunohistochemical scores using the upright microscope (Nikon eclipse 80i). The immunostaining was scored according to the German immunoreacted score and evaluated by two pathologists.^18^ Intensity was scored as 0 (negative), 1 (weak), 2 (moderate), and 3 (strong). Scores representing percentage of tumor cells positively stained were 0 (<5%), 1 (5%‐25%), 2 (25%‐50%), 3 (50%‐75%), or 4 (>75%).

### 2.5 DNA preparation and bisulfite conversion

Following the manufacturer's instructions, genomic DNA was extracted from HCC tissues and matched normal tissues using the QIAmp DNA Mini Kit (QIAGEN, Hilden, Germany). The concentrations of extracted DNA were measured by a NanoDrop 2000 (Thermo, Wilmington, USA). Genomic DNA (500 ng‐1 μg) was converted by sodium bisulfite according to the manufacturer's protocol of the EpiTect Fast DNA Bisulfite Kit (QIAGEN).

### 2.6 Gene bioinformatics and primer design

We obtained the information of *DNAH17* from the UCSC genome database (http://genome.ucsc.edu/). To identify the possible CpG sites, the CpG Island Finder of DBCAT software (http://dbcat.cgm.ntu.edu.tw/) was used to scan the sequences from chr17:76414778 to chr17:76573476. The primers used for quantitative methylation analysis of this gene were as follows: amplicon 1, forward primer aggaagagagGGGTTTTTTTGAGTTTTTGATTTTT and reverse primer cagtaatacgactcactatagggagaaggctATTTATAACAACCTACAATTCCCCA; amplicon 2, forward primer aggaagagagGGTTATTATAGGTGGTAGGGAGTGG and reverse primer cagtaatacgactcactatagggagaaggctCAACTAAAAAAACCCCAAACCTATT. A total of 15 CpG sites and 18 CpG sites were included in amplicon 1 and amplicon 2, respectively.

To explore the potential relationship between expression levels and methylation levels of *DNAH17*, we proceeded to query copy number, RNA‐seq and methylation data of this gene from the TCGA dataset. Moreover, three online networks, the Gene Expression Profiling Interactive Analysis website (GEPIA, http://gepia.cancer-pku.cn), Oncomine website (https://www.oncomine.org/), and cBioportal (http://www.cbioportal.org/), which analyzes bioinformation based on public databases, were also used to obtained more data.

### 2.7 Mass array quantitative methylation analysis

We used the Sequenom EpiTYPER assay to detect the methylation levels of the converted genomic DNA. This process includes three main steps: PCR amplification, SAP cleanup, and T cleavage. To verify the efficiency of PCR amplification, gel electrophoresis was performed after SAP cleanup. T cleavage was processed after confirming high efficiency of amplification. The products were then transferred to a SpectroCHIP® array and analyzed on the MassARRAY® Analyzer 4 instrument. We randomly picked 12 pairs of HCC and adjacent liver tissue samples for repeated experiments to verify the consistency of the experiment. The results of 24 DNA samples yielded a highly consistent result (*R* ^2^ = 0.95).

### 2.8 Gene copy number assay

*DNAH17* gene (Applied Biosystems, Cat #4400291) copy number was determined by TaqMan gene copy number assay in primary HCC and their corresponding normal tissues. The selected probe was located at amplicon 1. RNaseP gene (Applied Biosystems, Cat #4403326) was used as a standard reference gene. The copy number of target gene was analyzed and calculated by CopyCaller™ Software 2.1 (Applied Biosystems, Waltham, MA, USA). Predicted Ct values were used for the further analysis.

### 2.9 Statistical analysis

SPSS 20.0 version was used to perform the statistical analysis (IBM, Armonk, NY, USA). Given the abnormal distribution of the methylation levels in each CpG sites and the expression data, nonparametric Wilcoxon signed rank test was conducted to compare the methylation and expression differences and between HCC tissues and matched normal tissues. Paired two‐tailed Student's *t* test was conducted to compare the IHC score differences between HCC tissues and matched normal tissues. Also, paired two‐tailed Student's *t* test was used in decitabine treatment assay. Correlation between methylation‐level differences and clinicopathological characteristics were evaluated by linear regression analysis, adjusted with age and gender. Correlations between the methylation levels of each CpG sites were assessed by Pearson correlation analysis. Linear regression analysis was used to explore the correlation between copy number status and methylation levels, and the methylation differences among different CNV groups were calculated by nonparametric Mann‐Whitney test. We also conducted ROC curve to assess the methylation level of *DNAH17* as a predictive biomarker, and the discriminatory capacity was evaluated by calculating the area under curve (AUC). In general, a useless test has an AUC of 0.5, while an ideal test (one that has zero false negatives and zero false positives) has an AUC of 1.0. A *P* value <0.05 was considered as statistically significant.

## 3 RESULTS

### 3.1 Patient characteristics

The clinical and pathological characteristics of the patients are listed in Table 1. A total of 163 patients were included in this study. The male‐to‐female ratio was 138:25, and 137 patients had HBV‐related HCC. Thirty‐four patients consumed more than 50 grams per day (g/d) of alcohol. Ninety‐three patients were AFP positive, and 17 patients were CEA positive. For the clinicopathological characteristics, 21 patients presented with tumor thrombus and 17 patients had multiple liver tumors. Liver cirrhosis was found in 56 patients, and satellite tumor was detected in 44 patients. Most of the patients (89.6%) in this study were classified as stage III according to the staging system of AJCC.

**Table 1:** Association between methylation levels (%) of *DNAH17* gene and clinicopathological parameters

Parameters	Number	Mean methylation level of amplicon 1 (%)			Mean methylation level of amplicon 2 (%)		
		Normal	Tumor	*P*	Normal	Tumor	*P*
Gender							
Female	25	83.8	71.9	0.0076**	86.0	79.4	0.0120*
Male	139	84.8	55.9		83.8	68.2	
Age							
≤55	105	84.2	62.4	0.0406*	84.3	71.6	0.1632
＞55	58	85.2	52.7		83.8	67	
Diabetes							
Yes	14	87.9	58.1	0.3685	82.1	66.3	0.6084
No	144	84.3	62.3		84.5	70.5	
AFP							
＜20	70	84.2	49.9	0.0010**	84.4	65.8	0.0334*
≥20	93	84.8	65.5		84.0	73.1	
CEA							
≤5	146	84.2	59.4	0.7844	84.1	69.4	0.1173
＞5	17	87.4	52.6		84.5	74.4	
HBV							
Yes	137	84.6	57.9	0.2032	84.1	69.5	0.0769
No	25	85.9	61.7		84.5	71.5	
Alcohol habit							
Yes	34	85.4	55.6	0.8151	85.6	69	0.8239
No	129	82.8	60.1		83.6	70.3	
Tumor number							
1	145	84.3	59.8	0.0862	84.3	70.4	0.4471
2	12	88.2	43.5		82.1	64.8	
3	5	83.8	43.2		86.3	64	
Liver cirrhosis degree							
No	102	84.2	60.7	0.0648	85.2	73.1	0.0185*
Mild	30	86.3	46.9		84.6	62.5	
Moderate	24	84.2	61.8		81.9	66.7	
Severe	2	80.6	81.1		55.0	52.0	
Fibrous capsule							
Existed	107	84.5	53.6	0.0008**	85.1	68.6	0.3316
No	56	84.8	70.7		82.4	72.4	
Tumor necrosis							
Yes	124	83.8	56.1	0.0370*	83.7	69.3	0.6477
No	39	87.3	67.0		85.7	71.7	
Satellite tumor							
Yes	44	84.5	65.8	0.1202	82.0	73.5	0.2284
No	117	84.6	56.5		85.2	68.6	
Microvascular invasion							
Yes	57	83.1	60.7	0.6549	81.7	70.0	0.8396
No	106	85.3	58.0		85.6	69.8	
Tumor thrombus							
Yes	21	81.9	53.3	0.4415	77.5	58.5	0.0063**
No	142	84.8	59.0		85.4	71.5	
TNM Stage							
I‐II	17	84.0	59.1	0.2744	86.5	69.5	0.7876
III‐IV	146	84.7	58.7		83.9	69.9	

* *P* < 0.05;

** *P* < 0.01.

### 3.2 Methylation levels of *DNAH17* gene in HCC tissues and corresponding normal tissues

The location of *DNAH17* is chr17:76419778‐76573476, which belongs to 17q25.3 (Figure 1B). According to the UCSC genome database, there are three different transcripts of *DNAH17* gene, and one of these transcripts was non‐coding RNA. Therefore, we selected two amplicons close to the promoter regions of the other two isoforms (Figure 1C) to determine the methylation status of *DNAH17* in HCC. Amplicon 1, which yielded a 440 bp fragment, was near the promoter region of isoform 1 (chr17: 76445299‐76445756). Amplicon 2 was located at number 69 exon, covering the transcription start site (TSS) of isoform 2 (chr17: 76445299‐76445756). Fifteen CpG sites were identified in amplicon 1, and 12 sites (CpG3.4.5.6, CpG8.9, CpG10.11, CpG12, CpG13, CpG14, and CpG15) were successfully genotyped (Figure 1D). CpG3, CpG4, CpG5, and CpG6 were located close to each other and were genotyped as an integral, as were CpG8.9 and CpG10.11. A total of 18 CpG sites were identified in amplicon 2, and 13 CpG sites (CpG1, CpG2, CpG3.4, CpG5, CpG7, CpG10, CpG11.12, CpG13, CpG14, CpG15, CpG16, CpG17, and CpG18) were successfully genotyped (Figure 1D). Pearson correlation analysis showed that the methylation levels of each site were significantly correlated in amplicon 1 and amplicon 2, respectively (Tables S1 and S2).

**Figure 2:**
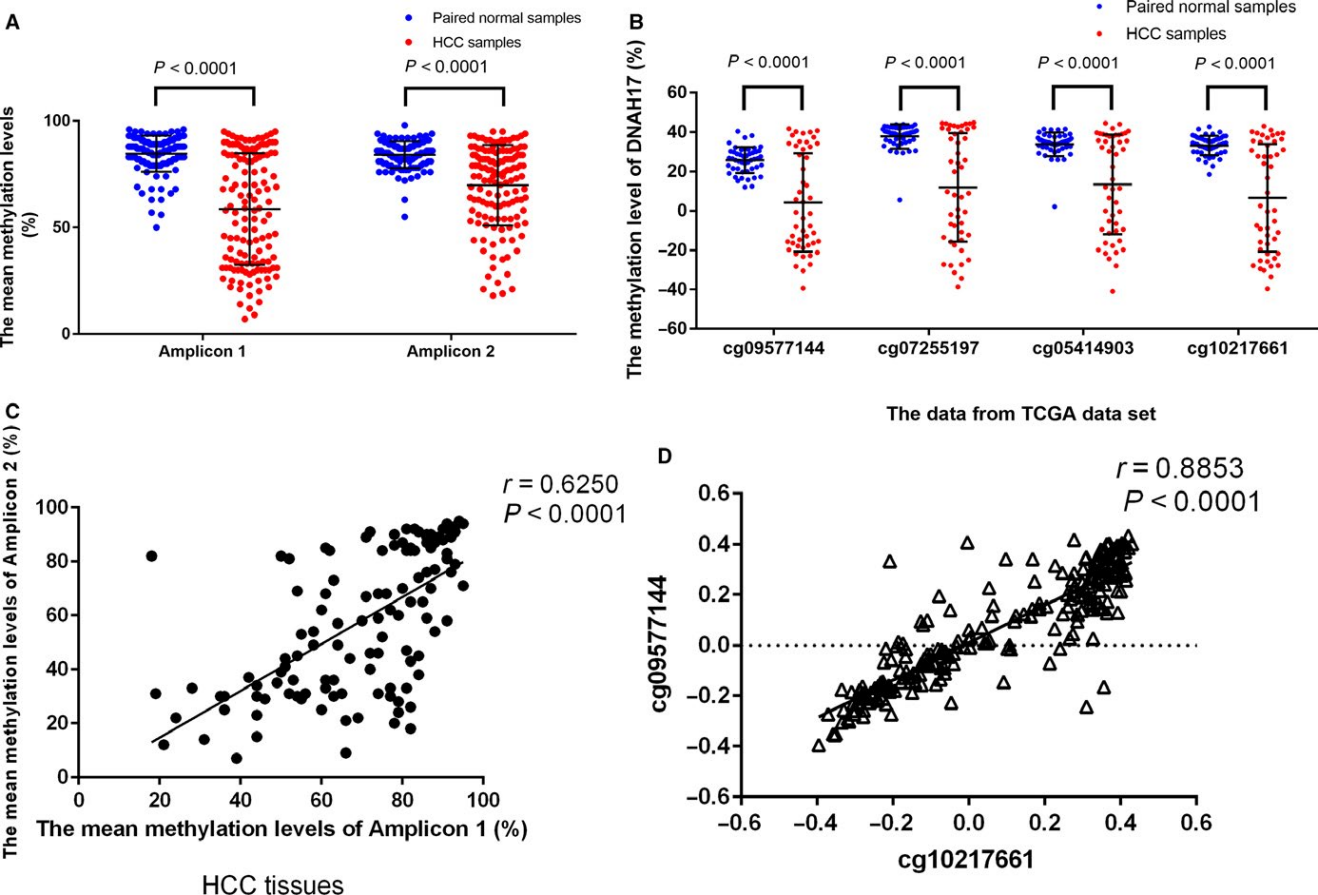
The methylation alternations of *DNAH17* in HCC. The mean methylation levels and standard deviation are shown as bar‐and‐whiskers plots (center line, mean; error bars, SD). A, The mean methylation levels were significantly decreased in 147 HCC tumor tissues in amplicon 1 and amplicon 2 of DNAH17 (Wilcoxon signed rank test). B, The methylation of the CpG sites close to the two studied amplicons decreased in 50 HCC samples from TCGA data set (Wilcoxon signed rank test). C, The correlation between mean methylation levels of amplicon 1 and amplicon 2 (Pearson correlation analysis). D, Correlation of the methylation status between cg00577144 site and cg10217661 site from TCGA data set (Pearson correlation analysis)

In amplicon 1, the methylation levels of the 12 CpG sites were significantly decreased in HCC samples compared to paired normal tissues (Table 2). In amplicon 2, we also observed the hypomethylation status of 11 CpG sites in HCC tissues (Table 2). The differences in methylation levels between tumor tissues and matched normal tissues ranged from 24.5% to 35.4% in amplicon 1, and from 2.4% to 25.1% in amplicon 2 (Table 2). The methylation levels of all CpG sites in amplicon 1 were found to be decreased more than 20%. The methylation levels of five CpG sites (CpG3.4, CpG5, CpG15, and CpG16) were found to be down‐regulated more than 20% in amplicon 2. In amplicon 1, the mean methylation level in the tumor tissues (58.7%) was significantly lower than that in the paired normal tissues (84.6%, *P* < 0.0001, Figure 2A). In amplicon 2, the mean methylation level was also decreased in the tumor tissues (69.9%) compared to the paired normal tissues (84.5%, *P* < 0.0001, Figure 2A). Moreover, our results were consistent with the data from the TCGA data set. Three CpG sites (cg09577144, cg07255197, and cg05414903) were located very close to amplicon 1. One CpG site (cg10217661) was close to amplicon 2. All four CpG sites were found to exhibit hypomethylation status in HCC tissue (Figure 2B).

**Table 2:** Methylation status (%) of *DNAH17* in amplicons in HCC patients

Amplicon 1					Amplicon 2				
CpGs	Group	Mean (%)	△Mean (%)	*P* value	CpGs	Group	Mean (%)	△Mean (%)	*P* value
CpG_3.4.5.6	Normal	93.9	24.5	<0.0001	CpG_1	Normal	46.5	−4.0	0.0925
	Tumor	69.4				Tumor	50.5		
CpG_8.9	Normal	94.9	27.2	<0.0001	CpG_2	Normal	87.8	14.3	<0.0001
	Tumor	67.7				Tumor	73.4		
CpG_10.11	Normal	90.5	35.4	<0.0001	CpG_3.4	Normal	94.6	23.3	<0.0001
	Tumor	55.1				Tumor	71.3		
CpG_12	Normal	67.5	27.2	<0.0001	CpG_5	Normal	79.7	21.4	<0.0001
	Tumor	40.2				Tumor	58.3		
CpG_13	Normal	83.2	28.7	<0.0001	CpG_7	Normal	92.7	18.6	<0.0001
	Tumor	54.5				Tumor	74.0		
CpG_14	Normal	82.4	28.1	<0.0001	CpG_10	Normal	96.5	8.6	<0.0001
	Tumor	54.3				Tumor	87.9		
CpG_15	Normal	81.0	27.8	<0.0001	CpG_11.12	Normal	85.7	14.3	<0.0001
	Tumor	53.2				Tumor	71.4		
					CpG_13	Normal	87.8	14.3	<0.0001
						Tumor	73.4		
					CpG_14	Normal	83.0	15.0	<0.0001
						Tumor	68.0		
					CpG_15	Normal	91.1	25.1	<0.0001
						Tumor	66.0		
					CpG_16	Normal	85.2	20.0	<0.0001
						Tumor	65.2		
					CpG_17	Normal	99.7	2.7	<0.0001
						Tumor	96.9		
					CpG_18	Normal	43.5	‐0.4	0.7864
						Tumor	43.9		

**Figure 3:**
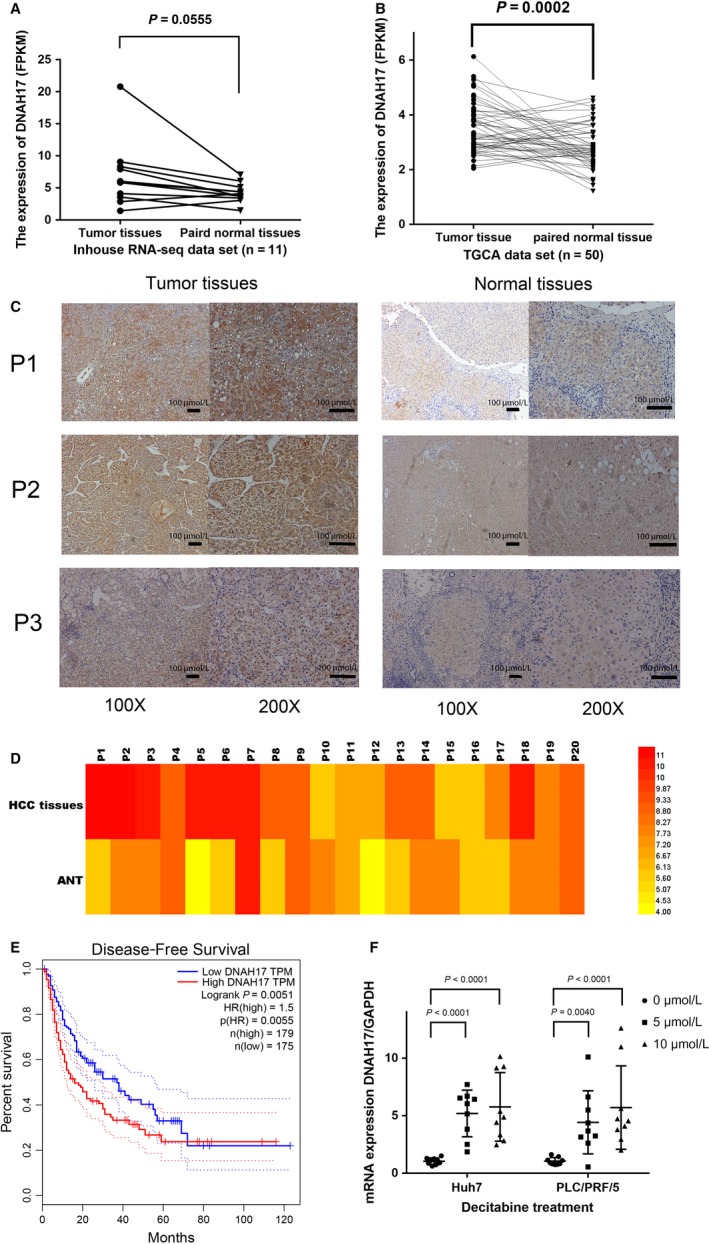
Expression of analyses of *DNAH17* mRNA and protein in HCC. A, The up‐regulated of *DNAH17* mRNA expression in HCC tissues from our previous RNA‐seq data set (n = 11, Wilcoxon signed rank test). B, Increased *DNAH17 *mRNA expression in HCC from TCGA database (n = 50, Wilcoxon signed rank test). C, Representative IHC image of *DNAH17* in HCC samples and ANT (P1: patient 1, P2: patient 2, P3: patient 3). D, Heatmap showed the difference for the IHC scores of 20 HCC patients (*P* = 0.0058, Paired two‐tailed Student's *t* test). E, The RFS data from the Gene Expression Profiling Interactive Analysis website (http://gepia.cancer-pku.cn), a network analyzing TCGA data online. Low expression of* DNAH17 *predicts a significantly better RFS rate (HR = 1.5, *P* = 0.0051). F, The expression of *DNAH17* was increased after decitabine treatment in Huh7 and PLC/PRF/5 cell lines (center line, mean; error bars, SD; Paired two‐tailed Student's *t* test

We further analyzed the correlation of the methylation status between amplicon 1 and amplicon 2 using Pearson correlation analysis. Interestingly, the methylation status of the two amplicons presented a powerfully positive correlation (*r* = 0.625, *P* < 0.0001, Figure 2C). We also found significant correlation between the three CpG sites close to amplicon 1 and the CpG site close to amplicon 2 (Figures 3D, S1 and S2).

### 3.3 Expression levels of *DNAH17* in HCC tissues and their paired adjacent non‐cancerous tissues

A transcriptome data set that contained 11 paired HCC samples was established in our previous study.^19^ In our RNA‐seq data set, the expression levels of *DNAH17 *showed a trend to increase in the tumor tissues (Figure 3A). We further proceeded to analyze the RNA‐seq data of 50 paired HCC samples in the TCGA data set. The expression levels were significantly higher in tumor tissues than in normal tissues (*P* = 0.0002, Figure 3B). We further used IHC to detect the protein changes in 20 paired HCC samples. We found 11 of 20 pairs of HCC samples (55%) presented high expression of *DNAH17 *in the tumor tissues compared with ANT, and *DNAH17* expression in HCC tissues was significantly higher than ANTs in most of HCC samples (*P* = 0.0058, Figure 3C,D).

In addition, the survival data from the GEPIA website demonstrate that the high *DNAH17* expression group predicts a worse disease‐free survival (HR = 1.5, *P* = 0.0051, Figure 3E). For overall survival, there was no significant difference between low‐expression patients and high‐expression patients (Figure S3).

To verify the expression of *DNAH17* was regulated by the methylation status, we selected two HCC cell lines, Huh7 and PLC/PRF/5, for decitabine treatment. The expression levels of *DNAH17* in both two HCC cell lines were increased after decitabine treatment for three days (Figure 3F).

### 3.4 The copy number variants of *DNAH17* in HCC samples

**Figure 4:**
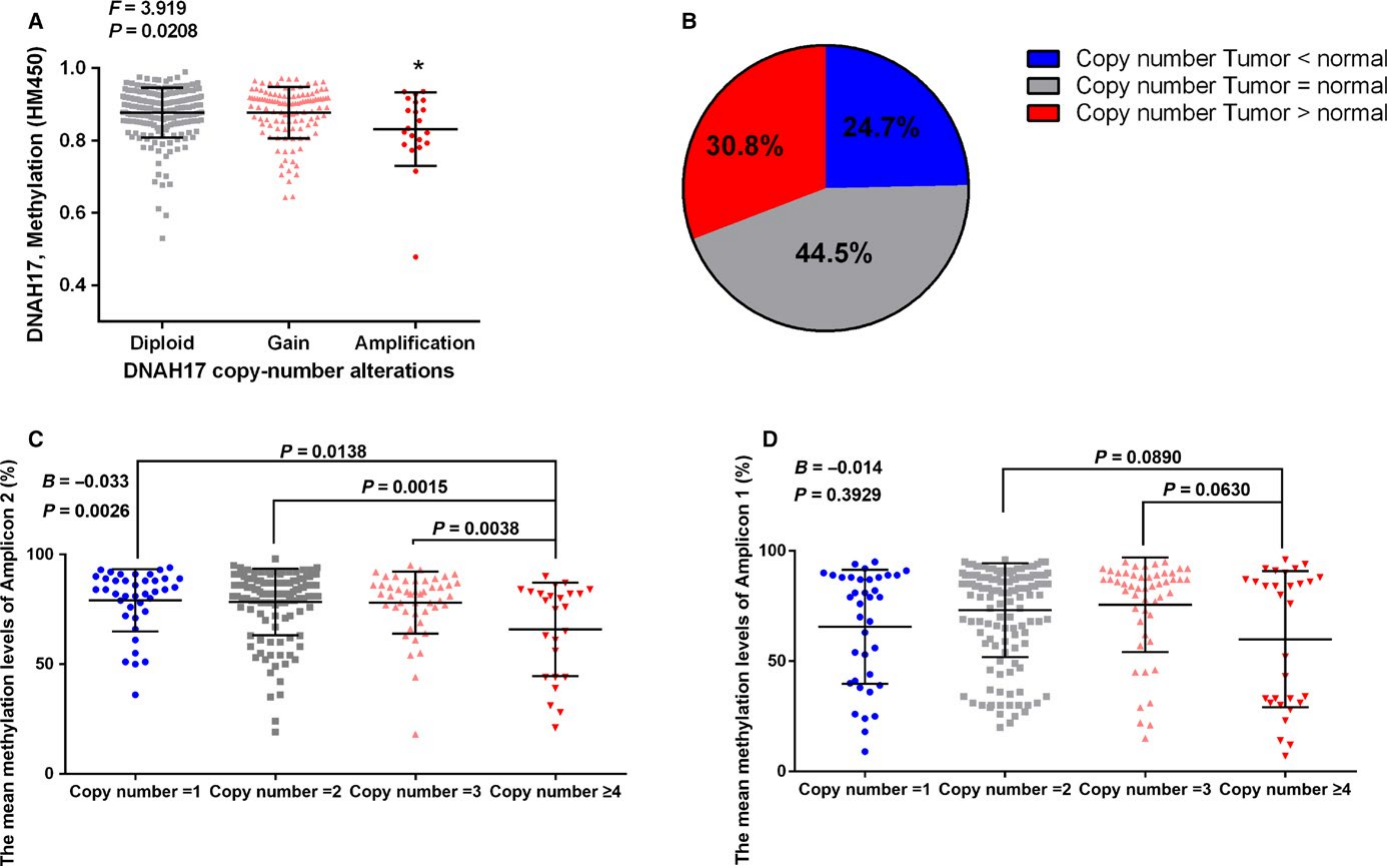
The methylation levels negative correlation with DNA copy number changes of *DNAH17 *(center line, mean; error bars, SD. Linear regression analysis and nonparametric Mann‐Whitney test). A, Data from TCGA data set. B, The proportion of copy number alteration in 163 HCC samples compared to ANT. The correlation between copy number and the mean methylation levels in (C) amplicon 1 and (D) amplicon 2

*DNAH17* copy number data and methylation‐level data were downloaded from Oncomine, a website analyzing TCGA data online. We observed that the methylation levels of *DNAH17* was significantly correlated with the CNV alternations, and the methylation values were down‐regulated in amplification patients compared to diploid patients (*P* = 0.0208, Figure 4A). To confirm the connection between copy number variants and the methylation of *DNAH17*, we used the TaqMan copy number assays to assess the copy number status of *DNAH17* in our cohort and found the copy number was increased in 30.8% HCC samples when compared to ANT (Figure 4B). In the amplicon 2, the copy number amplification samples always companied with hypomethylation status (Figure 4C). In the amplicon 1, although no significant association between methylation status and CNVs, the methylation levels in patients with no <4 copy numbers showed marginal difference to other patients with 2 or 3 DNAH17 copy number (Figure 4D). No significant correlation was found between copy number and clinicopathological features.

### 3.5 Correlation between the methylation of *DNAH17* and clinicopathological features

**Figure 5:**
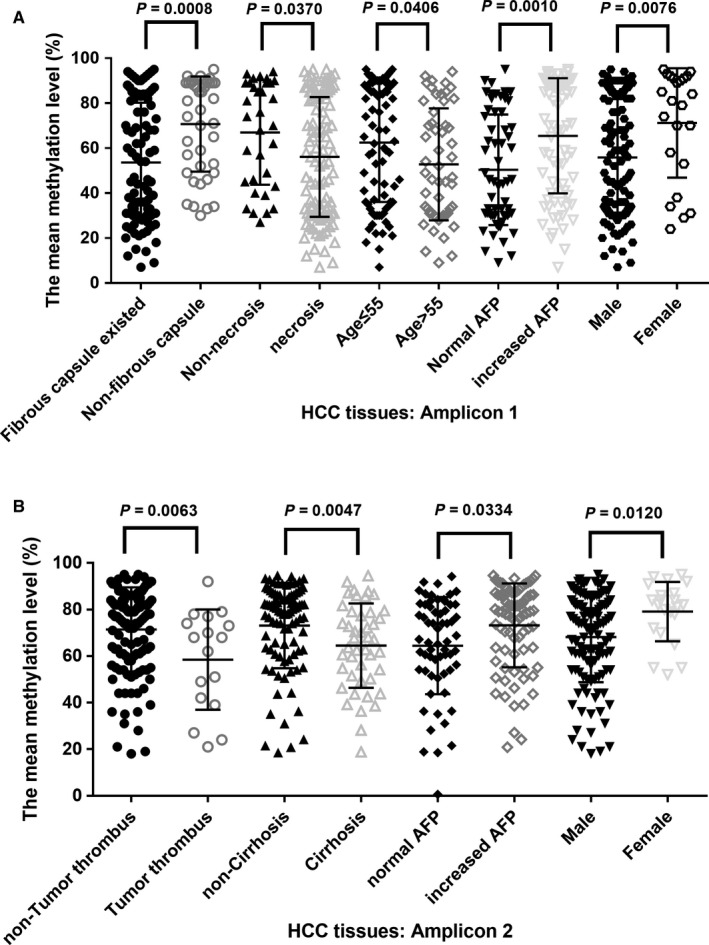
Association between methylation levels of *DNAH17* and clinicopathology feature in patients with HCC (center line, mean; error bars, SD; linear regression analysis). Correlation between methylation‐level differences and clinicopathological characteristics were evaluated by linear regression analysis, adjusted with age and gender. A, amplicon 1. B, in amplicon 2

To determine the role of *DNAH17* as a biomarker in HCC, the correlation between the methylation status of *DNAH17* and comprehensive clinicopathological features was analyzed (Table 1). Because most of the CpG sites were significantly correlated with each other, we calculated the mean values of the methylation level of all CpG sites to investigate the integrated effect of the selected amplicons. For amplicon 1, the hypomethylation status of *DNAH17* in HCC tissues was detected in patients with fibrous capsules (53.6% vs 70.7%, *P* = 0.0008, Figure 5A), tumor necrosis patients (56.1% vs 67.0%, *P* = 0.0370, Figure 5A), older patients (52.7% vs 62.4%, *P* = 0.0406, Figure 5A), AFP‐negative patients (49.9% vs 65.5%, *P* = 0.0010, Figure 5A), and male patients (56.1% vs 67.0%, *P* = 0.0076, Figure 5A). For amplicon 2, we observed that the mean methylation level of *DNAH17* in tumor tissues was significantly down‐regulated in patients with tumor thrombus (58.5% vs 78.5%, *P* = 0.0063, Figure 5B) and negative AFP (64.5% vs 73.2%, *P* = 0.0334, Figure 5B). The HCC samples with liver cirrhosis had lower *DNAH17* methylation levels than those without liver cirrhosis (non‐cirrhosis vs cirrhosis, 73% vs 64%, *P* = 0.0047, Figure 5B). A similar result was also found in paired normal tissue; the methylation level was significantly decreased (Figure S4). However, the methylation difference was obviously higher in tumor tissues (9%) than in paired normal tissues (2%). Hypomethylation status of *DNAH17* in HCC tissues was detected in male HCC patients (68.2% vs 79.1%, *P* = 0.0120, Figure 5B). As previously mentioned, the methylation levels of *DNAH17* in both amplicon 1 and amplicon 2 were significantly associated with age. We further analyzed the data from TCGA data set to verify the correlation. The same trends were detected in TCGA data set, even though the differences were marginally significant (Table S3).

### 3.6 ROC curve analysis of methylation levels in HCC and adjacent non‐cancerous tissues

**Figure 6:**
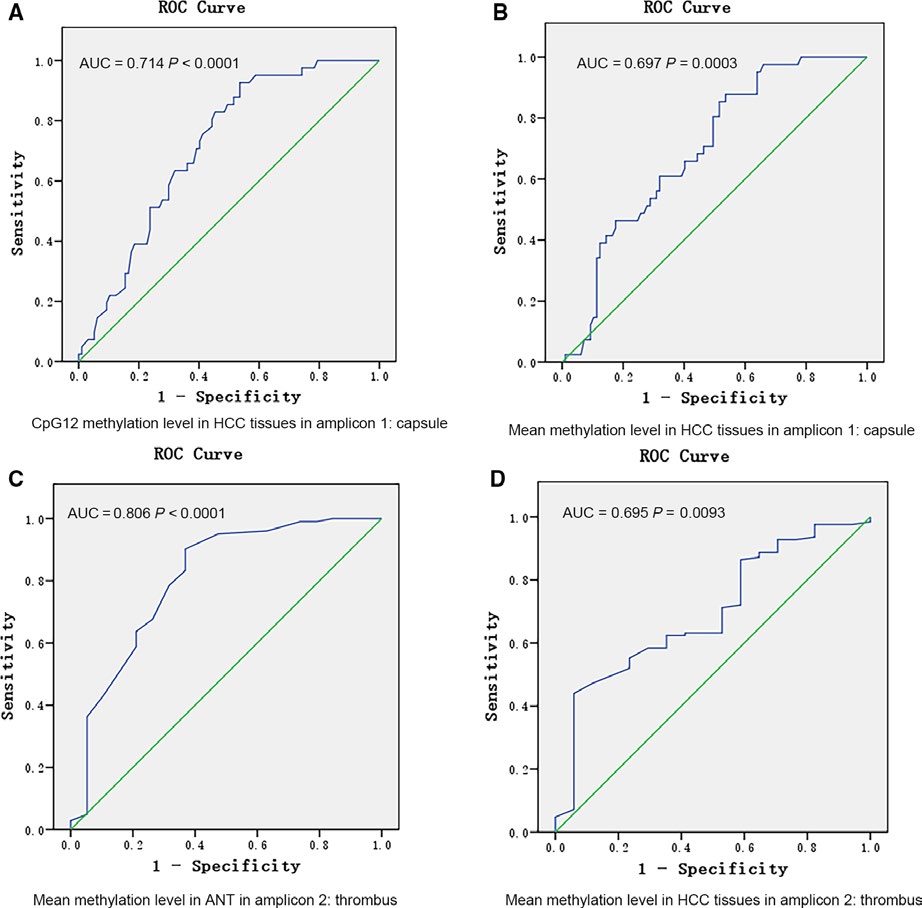
ROC curves analysis of the methylation levels in *DNAH17* for clinical features. A, ROC curve of the CpG12 methylation in amplicon 1 in tumor tissues for prediction the fibrous capsule. B, ROC curve of mean methylation level of all CpGs in amplicon 1 for prediction the fibrous capsule. C, ROC curve of mean methylation level of amplicon 2 in adjacent non‐cancerous tissues for prediction the tumor thrombus. D, ROC curve of mean methylation level of amplicon 2 in tumor tissues for prediction the tumor thrombus

To evaluate whether methylation levels of *DNAH17* can serve as a useful biomarker in HCC, ROC curves were plotted. The capacity of discrimination was assessed by calculating the AUC. We found that the methylation level of CpG12 in amplicon 1 (AUC = 0.714, *P* < 0.0001, Figure 6A) and the mean methylation level of amplicon 1 (AUC = 0.697, *P* = 0.0003, Figure 6B) discriminated HCC patients without fibrous capsules. ROC curve analysis showed that the mean methylation level of amplicon 2 in adjacent non‐cancerous tissues could efficiently discriminate HCC cases with tumor thrombus from those without tumor thrombus (AUC = 0.806, *P* < 0.0001, Figure 6C). However, the ability of discrimination of the mean methylation level in tumor tissues was lower than in adjacent non‐cancerous tissues (AUC = 0.695, *P* = 0.0093, Figure 6D).

## 4 DISCUSSION

DNA methylation, as one of the most widely studied epigenetic modifications, is crucial in various cancers. In the present study, hypomethylation status and overexpression of *DNAH17* were detected in HCC samples compared to paired normal tissues, suggesting that this epigenetic change of *DNAH17* might participate in HCC progression. Importantly, we found that *DNAH17* methylation was associated with gender, age, serum AFP values, liver cirrhosis, tumor fibrous capsule, tumor necrosis, and tumor thrombus. ROC curve analysis showed that hypomethylation status of *DNAH17 *in HCC patients could be a biomarker to predict the existence of fibrous capsule and tumor thrombus. In addition, we investigated the promoter region of two different isoforms in our experiment, which provide an intact view of this gene methylation status.

The protein encoded by*DNAH17* is a subunit of axonemal dynein, which is a basic structure for primary cilia. Many studies indicated that the primary cilia played an important role in embryonic development, cell differentiation, cell division, and tumor progression through Hedgehog (Hh) signaling.^20^, ^21^, ^22^ As we described above, loss of primary cilia is a common feature of malignant cells, whereas our results showed that the expression of *DNAH17* was up‐regulated in HCC tissues by hypomethylation. The paradox might observed be because the primary cilia are both positive and negative effectors of Hh signaling.^11^ Moreover, Wang et al^23^ reported that dynein axonemal heavy chain 8, a DNAH family protein, was overexpressed in prostate cancer tissues compared to normal prostate tissues and could promote cancer metastasis.

*DNAH17* is located at 17q25.3, which resides in an amplicon that is significantly associated with many cancers, including HCC.^24^, ^25^, ^26^, ^27^ It was reported that 17q25.1‐3 copy number gain was a prognostic marker for poor patient survival in HCC.^27^ In liver cancer, the methylation‐correlated expression genes and DNA copy number‐correlated expression genes are significantly co‐regulated.^5^ We then explored the methylation status and copy number of *DNAH17 *in our cohort and noted that a lower methylation level of HCC samples was observed in *DNAH17 *amplification patients compared with the rest patients, which consisted with the data from TCGA data set. This finding indicated that the *DNAH17* overexpression in HCC tissue was possibly regulated by the synergy of gene amplification and hypomethylation. However, we should realize that the qPCR method is not an ideal method to assess the copy number variant in tumor tissues because of the high heterogeneity of tumor. The different proportion of cancer cells in different tumor samples could also bring bias into our study.

It is well known that male patients are markedly predominant in morbidity and mortality in HCC.^28^ Shen et al^29^ explored genome‐wide DNA methylation profile changes in HCC and found that some CpG sites were significantly associated with gender. A similar phenomenon was also detected in our study. Lower methylation levels of *DNAH17* promoter in HCC samples were found in male patients. In AFP‐negative HCC patients, we found that the methylation level of *DNAH17* in HCC samples was lower than that in AFP‐positive HCC patients. Several studies also indicated that the methylation status of some HCC‐related genes in HCC tissues was associated with abnormal serum AFP level.^30^, ^31^ Furthermore, this finding encouraged us to explore whether the methylation detection of *DNAH17* gene in circulating tumor DNA (ctDNA) could be a sensitive biomarker for AFP‐negative HCC patients which would be useful for HCC early diagnosis. We also found the hypomethylation status of the *DNAH17* isoform 1 promoter was significantly associated with tumor necrosis and the existence of fibrous capsule. In both ANT and HCC tissues, the methylation differences of the *DNAH17* isoform 2 promoter between liver cirrhosis patients and non‐cirrhosis patients were significant. The hypomethylation status of *DNAH17 *isoform 2 was detected in liver cirrhosis tissues. However, the methylation difference between cirrhosis patients and non‐cirrhosis patients was 4.5 times higher in HCC tissues than in ANT. Therefore, we speculated that *DNAH17* is not only associated with liver cirrhosis, but also plays a more important role in oncogenesis under the liver cirrhosis background. The underlying mechanism needs to be confirmed in future studies.

Macrovascular invasion, including tumor thrombosis in the portal vein and hepatic vein, is a poor prognosis factor.^2^ However, not all tumor thrombosis in HCC patients could be detected preoperatively, which often leads to a dilemma during surgery. Here, we found that HCC patients with tumor thrombus were always accompanied by hypomethylation of *DNAH17* in the promoter of isoform 2, which implied that down‐regulated methylation of *DNAH17* might promote HCC metastasis. We further evaluated the potential values of *DNAH17 *methylation in our study for clinical diagnosis purposes. The discriminatory capacity of *DNAH17* methylation levels was evaluated to distinguish HCC patients with tumor thrombus through ROC curve analysis. Our findings showed that the hypomethylation status of *DNAH17* in both tumor tissues and adjacent non‐cancerous tissues could help discriminate HCC patients with tumor thrombus from those without tumor thrombus. More importantly, the AUC of the mean methylation level in paired normal tissues was more than 0.8. Our result indicated that the methylation status of *DNAH17* was a promising biomarker for tumor thrombosis, which could provide more information about HCC features in the preoperative biopsy and help clinicians generate an individual treatment strategy. Moreover, the significantly aberrant methylation of *DNAH17* in HCC tissues suggested that this gene could be a promising candidate for liquid biopsy in future research.

In summary, hypomethylation status of the *DNAH17 *gene was detected in HCC. The *DNAH17* overexpression in HCC tissue was possibly regulated by the synergy of gene amplification and hypomethylation. We revealed that methylation alterations in *DNAH17 *have a correlation with age, gender, serum AFP level, liver cirrhosis, tumor necrosis, fibrous capsule, and tumor thrombus. Importantly, ROC analysis demonstrated that hypomethylation status could be a promising biomarker to predict the existence of the tumor thrombosis. The underlying mechanism needs to be further confirmed.

## CONFLICT OF INTEREST

The authors declare no conflict of interest.
