# Supplementary material for: The association between methylation patterns of DNAH17 and clinicopathological factors in hepatocellular carcinoma
Source: Cancer Med. 2018 Dec 21;8(1):337–50. doi: 10.1002/cam4.1930 (PMC6346260; doi:10.1002/cam4.1930)
Supplement: Supplementary file 1 [file CAM4-8-337-s001.docx]

**Supplementary materials**

Supplementary table 1. The correlation of the methylation among the 12 genotyped CpG sites in amplicon 1.

| Correlation | | Pearson Correlation | | | | | | |
| --- | --- | --- | --- | --- | --- | --- | --- | --- |
|  |  | TCpG1 | TCpG2 | TCpG3.4 | TCpG5 | TCpG7 | TCpG10 | TCpG11.12 |
| P value | TCpG1 | 1 | 0.701 | 0.629 | 0.653 | 0.739 | 0.745 | 0.725 |
|  | TCpG2 | <0.0001 | 1 | 0.685 | 0.742 | 0.721 | 0.773 | 0.728 |
|  | TCpG3.4 | <0.0001 | <0.0001 | 1 | 0.888 | 0.745 | 0.801 | 0.850 |
|  | TCpG5 | <0.0001 | <0.0001 | <0.0001 | 1 | 0.722 | 0.804 | 0.859 |
|  | TCpG7 | <0.0001 | <0.0001 | <0.0001 | <0.0001 | 1 | 0.795 | 0.799 |
|  | TCpG10 | <0.0001 | <0.0001 | <0.0001 | <0.0001 | <0.0001 | 1 | 0.899 |
|  | TCpG11.12 | <0.0001 | <0.0001 | <0.0001 | <0.0001 | <0.0001 | <0.0001 | 1 |

Supplementary table 2. The correlation of the methylation among the 15 genotyped CpG sites in amplicon 2.

| Correlation | | Pearson Correlation | | | | | | | | | | | | |
| --- | --- | --- | --- | --- | --- | --- | --- | --- | --- | --- | --- | --- | --- | --- |
|  |  | TCpG1 | TCpG2 | TCpG3.4 | TCpG5 | TCpG7 | TCpG10 | TCpG11.12 | TCpG13 | TCpG14 | TCpG15 | TCpG16 | TCpG17 | TCpG18 |
| P value | TCpG1 | 1 | 0.280 | 0.236 | -0.022 | 0.189 | 0.257 | 0.386 | 0.280 | 0.031 | 0.070 | 0.162 | 0.041 | 0.673 |
|  | TCpG2 | <0.0001 | 1 | 0.809 | 0.605 | 0.590 | 0.628 | 0.688 | 1.000 | 0.642 | 0.711 | 0.736 | 0.405 | 0.231 |
|  | TCpG3.4 | 0.0040 | <0.0001 | 1 | 0.686 | 0.745 | 0.610 | 0.683 | 0.809 | 0.578 | 0.763 | 0.822 | 0.428 | 0.220 |
|  | TCpG5 | 0.7850 | <0.0001 | <0.0001 | 1 | 0.585 | 0.574 | 0.475 | 0.605 | 0.665 | 0.724 | 0.749 | 0.439 | -0.064 |
|  | TCpG7 | 0.0220 | <0.0001 | <0.0001 | <0.0001 | 1 | 0.518 | 0.679 | 0.590 | 0.599 | 0.665 | 0.680 | 0.373 | 0.291 |
|  | TCpG10 | 0.0011 | <0.0001 | <0.0001 | <0.0001 | <0.0001 | 1 | 0.623 | 0.628 | 0.594 | 0.521 | 0.622 | 0.401 | 0.168 |
|  | TCpG11.12 | <0.0001 | <0.0001 | <0.0001 | <0.0001 | <0.0001 | <0.0001 | 1 | 0.688 | 0.517 | 0.550 | 0.633 | 0.337 | 0.383 |
|  | TCpG13 | <0.0001 | <0.0001 | <0.0001 | <0.0001 | <0.0001 | <0.0001 | <0.0001 | 1 | 0.642 | 0.711 | 0.736 | 0.405 | 0.231 |
|  | TCpG14 | 0.701 | <0.0001 | <0.0001 | <0.0001 | <0.0001 | <0.0001 | <0.0001 | 0.000 | 1 | 0.719 | 0.720 | 0.444 | 0.074 |
|  | TCpG15 | 0.396 | <0.0001 | <0.0001 | <0.0001 | <0.0001 | <0.0001 | <0.0001 | <0.0001 | <0.0001 | 1 | 0.878 | 0.406 | 0.108 |
|  | TCpG16 | 0.046 | <0.0001 | <0.0001 | <0.0001 | <0.0001 | <0.0001 | <0.0001 | <0.0001 | <0.0001 | <0.0001 | 1 | 0.461 | 0.137 |
|  | TCpG17 | 0.615 | <0.0001 | <0.0001 | <0.0001 | <0.0001 | <0.0001 | <0.0001 | <0.0001 | <0.0001 | <0.0001 | <0.0001 | 1 | 0.026 |
|  | TCpG18 | <0.0001 | 0.004 | 0.008 | 0.427 | <0.0001 | 0.038 | <0.0001 | 0.004 | 0.366 | 0.190 | 0.092 | 0.748 | 1 |

Supplementary table 3. The normalized mean methylation levels (%) of DNAH17 in different gender patients from TCGA data set (n=49, Wilcoxon Signed Ranks test).

| Probe ID | gender | Number | Mean (%) | P value |
| --- | --- | --- | --- | --- |
| cg10217661 | Female | 56 | 8.27 | 0.1457 |
|  | Male | 90 | 1.73 |  |
| cg09577144 | Female | 56 | 8.75 | 0.0641 |
|  | Male | 90 | 1.39 |  |
| cg07255197 | Female | 56 | 18.45 | 0.0386 |
|  | Male | 90 | 11.42 |  |
| cg05414903 | Female | 56 | 17.33 | 0.1782 |
|  | Male | 90 | 13.04 |  |

**Supplementary Figure 1.** Correlation of the methylation status between cg07255197 sites and cg10217661 sites (Pearson correlation analysis).





**Supplementary Figure 2.** Correlation of the methylation status between cg05414903 sites and cg10217661 sites (Pearson correlation analysis).





**Supplementary Figure 3**. Low expression of DNAH17 tends to predict a better overall survival rate, but there is no statistical significance (HR=1.4, P=0.086, from GEPIA website).


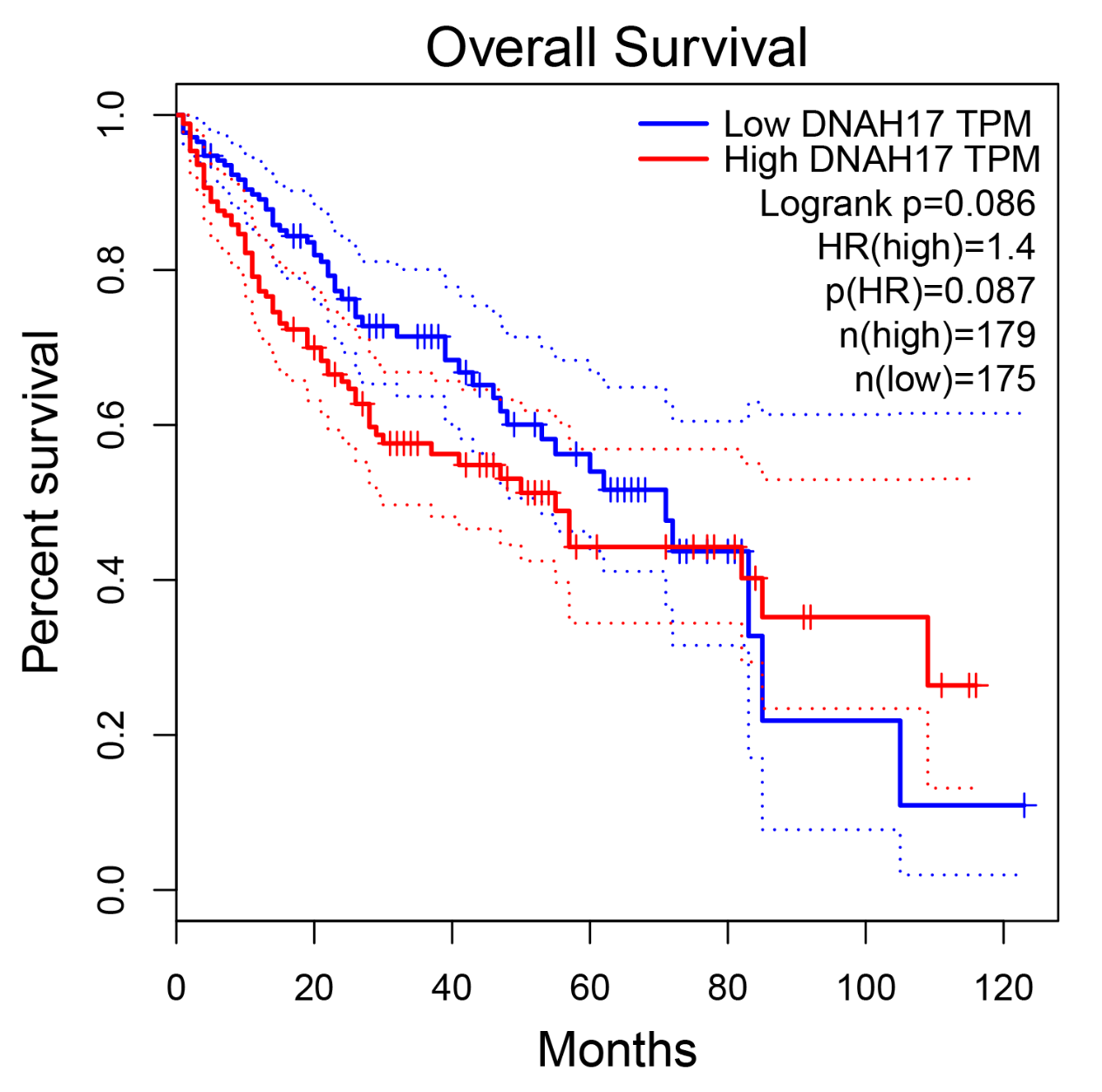


**Supplementary Figure 4**. Methylation level of DNAH17 was lower in liver cirrhosis ANT than in non-cirrhosis ANT. center line represented the mean value and error bars represented SD (Linear regression analysis).
